# Inhibition of angiogenesis and suppression of colorectal cancer metastatic to the liver using the *Sleeping Beauty *Transposon System

**DOI:** 10.1186/1476-4598-10-14

**Published:** 2011-02-10

**Authors:** Lalitha R Belur, Kelly M Podetz-Pedersen, Brent S Sorenson, Alice H Hsu, Josh B Parker, Cathy S Carlson, Daniel A Saltzman, S Ramakrishnan, R Scott McIvor

**Affiliations:** 1 Center for Genome Engineering, University of Minnesota, Minneapolis, MN 55455, USA; 2 Gene Therapy Program, Institute of Human Genetics, University of Minnesota, Minneapolis, MN 55455, USA; 3 Department of Genetics, Cell Biology and Development, University of Minnesota, Minneapolis, MN 55455, USA; 4 Department of Veterinary Population Medicine, University of Minnesota, St. Paul, MN 55108, USA; 5 Department of Surgery, University of Minnesota, MN 55455, USA; 6 Department of Pharmacology, University of Minnesota, Minneapolis, MN 55455, USA

## Abstract

**Background:**

Metastatic colon cancer is one of the leading causes of cancer-related death worldwide, with disease progression and metastatic spread being closely associated with angiogenesis. We investigated whether an antiangiogenic gene transfer approach using the *Sleeping Beauty *(SB) transposon system could be used to inhibit growth of colorectal tumors metastatic to the liver.

**Results:**

Liver CT26 tumor-bearing mice were hydrodynamically injected with different doses of a plasmid containing a transposon encoding an angiostatin-endostatin fusion gene (Statin AE) along with varying amounts of SB transposase-encoding plasmid. Animals that were injected with a low dose (10 μg) of Statin AE transposon plasmid showed a significant decrease in tumor formation only when co-injected with SB transposase-encoding plasmid, while for animals injected with a higher dose (25 μg) of Statin AE transposon, co-injection of SB transposase-encoding plasmid did not significantly affect tumor load. For animals injected with 10 μg Statin AE transposon plasmid, the number of tumor nodules was inversely proportional to the amount of co-injected SB plasmid. Suppression of metastases was further evident in histological analyses, in which untreated animals showed higher levels of tumor cell proliferation and tumor vascularization than animals treated with low dose transposon plasmid.

**Conclusion:**

These results demonstrate that hepatic colorectal metastases can be reduced using antiangiogenic transposons, and provide evidence for the importance of the transposition process in mediating suppression of these tumors.

## Background

Carcinoma of the colon is the second most common cause of cancer-related death in the United States and other developed countries [1]. The primary cause of mortality is dissemination of the disease to secondary sites, with the liver being the primary, and most critical, organ for development of metastasis [2,3]. Liver resection is the only effective treatment to facilitate a potential cure. However, less than 10% of patients are eligible for surgery, since they present with advanced or disseminated disease due to the absence of early diagnostic symptoms [2-4].

Tumor neovascularization plays a critical role in colorectal cancer progression, and increased angiogenesis has been associated with poor prognosis and relapse of colorectal disease [5,6]. There are several small molecule inhibitors of angiogenesis currently in clinical trials [7]. The anti-VEGF antiangiogenic antibody bevacizumab is now used clinically as a first line treatment in combination with standard first and second-line chemotherapy regimens for treatment of metastatic colorectal cancer, conferring a significant increase in survival time (20-25 months) [8,9]. However, antiangiogenic factors have a cytostatic rather than cytotoxic effect, therefore requiring continuous and possibly lifelong administration of the recombinant protein [10,11]. Introduction of sequences encoding antiangiogenic gene products is an alternate approach to achieve continuous and sustained expression of angiostatic factors in neoplastic tissue, thus counteracting tumor-induced angiogenesis.

Both viral and non-viral vector systems have been tested for potential therapeutic gene transfer against colorectal cancer. Viral vectors have been used by most investigators for gene delivery, due to the higher efficiency of gene transfer compared to non-viral systems. Viral vector types that have been used to deliver antiangiogenic genes for therapy of colorectal cancer include adenoviral vectors [12-15] and adeno-associated viral (AAV) vectors [16], and non-viral vectors include HVJ cationic liposomes and naked plasmid DNA. HVJ-cationic liposomes were shown to be effective in inhibiting angiogenesis by repeat intratumoral injections of vector encoding mouse macrophage metalloelastase in a subcutaneous model of colorectal cancer [17]. Uesato et al expressed angiostatin and endostatin in subcutaneous tumors after repeated low-voltage electroporation and achieved decreased tumor growth [18]. More recently, Wen et al reported hydrodynamic plasmid injection to express NK4 in a hepatic model of liver metastasis, with successful inhibition of tumor formation [19,20]. Non-viral anti-angiogenic gene delivery has thus, been used successfully, with therapeutic benefits in inhibiting the growth of colorectal tumors, but the duration of effectiveness is constrained by the transient period of gene expression.

The *Sleeping Beauty *(SB) transposon system combines the advantages of non-viral plasmid-based vector systems with the integrative capabilities of some viral vectors. This plasmid-based vector system provides prolonged expression of the transgene through integration into the host chromosome, thereby circumventing the need for repeated administration of the therapeutic gene [21]. The SB transposon system has been successfully used to transfer genes into a variety of cell types [22-25], including neoplastic tissue [26-28]. This system consists of 2 components; a transposon, comprising a gene of interest flanked by indirect repeat sequences, and the synthetic SB transposase, which catalyzes excision and integration of the gene into genomic DNA.

In the present study, the SB transposon system was used to achieve transfer of antiangiogenic genes into tumor-bearing animals. We investigated the antitumor effects of a transposon vector that encodes an angiostatin-endostatin fusion gene (Statin AE), administered in a CT26 mouse model of colorectal cancer metastatic to the liver. Statin AE transposon administration was associated with a significant antitumor effect as gauged by inhibition of tumor growth, and reduction in tumor vasculature. A dose-dependent requirement for SB transposase-encoding plasmid at lower doses of Statin AE transposon was observed, implicating the importance of transposition and stable Statin AE expression in low substrate (transposon) conditions such as those likely to be achieved in large animals or humans. These results demonstrate the potential effectiveness of the SB transposon system for therapeutic antiangiogenic gene transfer in metastatic colorectal cancer.

## Results

### Hepatic Gene Delivery and Localization of Gene Expression

**Figure 1:**
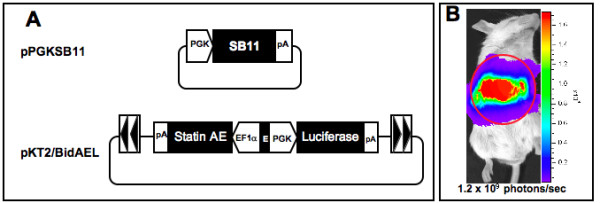
**Gene delivery and expression of firefly luciferase in the liver**. **A**, Plasmid constructs used in gene delivery to the liver. PGK, phosphoglycerate kinase; pA, SV40 polyadenylation signal; SB11, *Sleeping Beauty *11 transposase gene; EF1-α, elongation factor1-α; E, CpG-free synthetic enhancer; inverted arrowheads, T2 transposon inverted repeats. **B**, Representative mouse from the group that received 10 μg transposon and 10 μg SB plasmids showing gene delivery and expression in the liver 24 hrs following hydrodynamic gene delivery.

There have been several studies reported in which vectors containing either angiostatin or endostatin resulted in significant tumor regression upon administration to tumor bearing mice. In addition, combined treatment of tumor bearing mice with vectors encoding both angiostatin and endostatin resulted in an additive effect in tumor growth inhibition when compared to treatment using either of these agents alone [29-31]. The StatinAE gene, which is a fusion of murine angiostatin and endostatin genes, has been demonstrated to confer significant inhibition of subcutaneously implanted human glioblastoma and melanoma xenografts [27,32]. We therefore constructed a *Sleeping Beauty *transposon plasmid containing the StatinAE and firefly luciferase genes regulated by a bidirectional promoter (pKT2/BidEAL, Figure 1A). In this construct a luciferase transgene is transcriptionally regulated by the phosphoglycerate kinase (PGK) promoter, while in the opposite orientation a CpG free enhancer/elongation factor 1-alpha (EF1-α) promoter regulates expression of an angiostatin-endostatin fusion gene [32]. To form the transposon, the transcriptional cassettes are flanked by *Sleeping Beauty *T2 transposon inverted terminal repeats [33].

**Figure 2:**
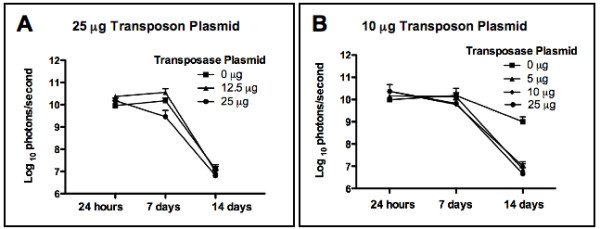
**Time course and levels of luciferase expression following hydrodynamic gene delivery**. Animals were injected with plasmids as indicated below, and imaged at various time points for in vivo luciferase gene expression (mean +/- positive SD). **A**, 25 μg high dose transposon plasmid; **B**, 10 μg low dose transposon plasmid.

Murine CT26 colon carcinoma is a transplantable tumor that shows strong hypervascularization and is an effective model for evaluation of an angiostatic, antitumor approach. Balb/c mice were injected with CT26 colorectal tumor cells intrasplenically, subsequently allowing the cells to seed into the liver and initiate localized tumor growth. Two days following tumor seeding, animals were hydrodynamically injected (see Materials and Methods) with 10 μg (low dose) or 25 μg (high dose) of pKT2/BidAEL along with either 0, 5, 10, 12.5 or 25 μg of SB transposase plasmid. Plasmid delivery to the liver was assessed by in vivo bioluminescence imaging of luciferase expression at 24 hours, 1 week and two weeks post-injection. Figure 1B depicts a representative animal with localized hepatic expression of luciferase imaged at 24 hours after plasmid injection. Levels of expression in the high dose and low dose transposon groups are depicted in Figures 2A and 2B respectively. All animals, regardless of presence or absence of SB plasmid, showed similar levels of expression, with the highest levels observed at 24 hours and 1 week post-injection, ranging from 6 × 10^9 ^to 5 × 10^10 ^photons/sec. At 2 weeks post-injection, luciferase expression was markedly reduced to levels that were approximately 1000-fold lower (mean of 1.2 × 10^7 ^photons/sec). Levels of luciferase expression in both the high and low dose transposon groups were similar, and both groups exhibited a similar time course of expression over two weeks. These results confirm hepatic delivery and subsequent transgene expression of the therapeutic pKT2/BidAEL plasmid.

### Effect of antiangiogenic transposon gene therapy on tumor load

**Figure 3:**
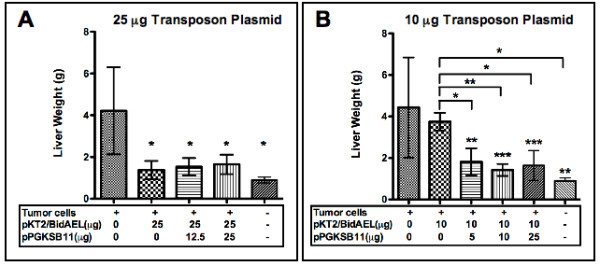
**Decrease in liver weights following antiangiogenic transposon gene therapy**. Animals were sacrificed 21 days post-tumor injection, livers were extracted and weighed, to gauge effect of treatment on tumor load (mean +/- SD). Transposon (pKT2/BidAEL), and transposase (pPGKSB11) plasmid DNA amounts infused into animals are summarized below the bar graph. **A**, 25 μg high dose transposon plasmid; **B**, 10 μg low dose transposon plasmid. *P < 0.05; **P < 0.005, ***P < 0.0005

**Figure 4:**
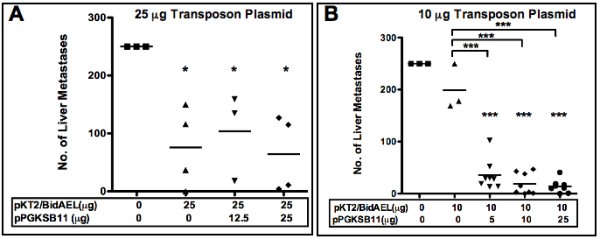
**Effects of antiangiogenic transposon gene therapy on liver metastases**. Livers were extracted from sacrificed animals and tumor nodules on the liver were enumerated. Transposon (pKT2/BidAEL), and transposase (pPGKSB11) plasmid DNA amounts infused into animals are summarized below the graph. **A**, 25 μg high dose transposon plasmid (bar indicates mean of n = 3-4); **B**, 10 μg low dose transposon plasmid, (bar indicates mean of n = 8). *P < 0.05; ***P < 0.0005

Animals were sacrificed 21 days after tumor infusion, and the effect of antiangiogenic transposon treatment on tumor burden was gauged by total liver weight (Figures 3A and 3B) as well as reduction of the number of tumor metastases (Figures 4A and 4B). The mean liver weight of untreated tumor bearing animals (4.2 +/- 2.1 g) was significantly higher (P < 0.01) than that of each of the groups of antiangiogenic transposon-treated tumor bearing animals, in which the mean weights ranged from 1.37 to 1.65 g, as well as the non-tumor bearing control group, which had a mean liver weight of 0.9 g. There was no significant difference in liver weights between groups that were co-injected with either 0, 12.5, or 25 μg of SB transposase plasmid along with 25 μg of Statin AE transposon or between any of these groups and the non-tumor bearing control group (Figure 3A).

Tumor-bearing animals that were injected with low dose (10 μg) Statin AE transposon plasmid alone had a mean liver weight of 3.75 +/- 0.43 g, which was not significantly lower than that of the untreated tumor-bearing control group (4.4 +/- 2.4 g). However, animals that were co-injected with either 5, 10, or 25 μg of SB transposase plasmid had mean liver weights ranging from 1.4 +/- 0.3 g to 1.8 +/- 0.7 g and these were significantly lower (P < 0.0005) than untreated tumor-bearing control animals and tumor-bearing animals administered 10 μg transposon plasmid alone (Figure 3B).

In all groups of animals treated with a high dose (25 μg) of Statin AE transposon DNA, there was a significant reduction in the number of liver tumor nodules in comparison with that of untreated tumor bearing animals (P < 0.05), whether or not SB transposase plasmid was co-injected (Figure 4A). In addition, the results were similar regardless of dosage of SB transposase.(12.5 or 25 μg). However, in animals treated with a low dose (10 μg) of Statin AE transposon alone (Figure 4B), antitumor effectiveness was observed only in animals co-administered SB transposase-encoding DNA. Infusion of 10 μg Statin AE transposon alone caused only a minimal (nonsignificant) reduction in liver tumor nodules compared to untreated tumor-bearing animals. In contrast, there was a significant decrease in metastases (P < 0.0001) observed in animals co-infused with 5 μg (36 +/- 29), 10 μg (19 +/- 20), or 25 μg (14 +/- 12) SB plasmid, i.e a trend toward decreased nodule formation with increasing doses of SB transposase plasmid.

### Effect of antiangiogenic transposon treatment on histological indices of metastatic tumors

**Figure 5:**
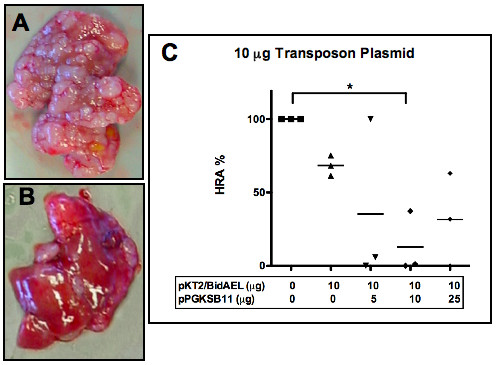
**Effects of transposon antiangiogenic therapy on macroscopic appearance and tumor hepatic replacement area (HRA) of resected livers**. Livers from sacrificed animals were evaluated macroscopically for clinical appearance, and histologically, for replacement of hepatic tissue by tumor tissue. Transposon (pKT2/BidAEL), and transposase (pPGKSB11) plasmid DNA amounts infused into animals are summarized below the graph. **A**, Liver from untreated tumor bearing control animal; **B**, Liver from animal treated with 10 μg low dose transposon + 25 μg SB transposase; **C**, % HRA in cohorts of animals treated with low dose of transposon plasmid (bar indicates mean of n = 3 per group). *P < 0.05

To evaluate the effect of anti-angiogenic gene therapy, animals were sacrificed at 3 weeks post-injection and livers were analyzed histologically for the presence and extent of replacement of normal liver by tumor tissue. Livers of untreated tumor bearing mice were enlarged and contained multiple, coalescing pale nodules that replaced nearly all of the normal hepatic parenchyma (Figure 5A), while the livers of animals treated with Statin AE transposon plasmid were smaller in size and contained tumor nodules that replaced a much lower volume of tissue (Figure 5B).

The hepatic replacement area (HRA) (defined as the percentage of normal hepatic tissue that has been displaced by metastatic tumor) was assessed morphometrically in histological sections for the low dose transposon mice, and is summarized in Figure 5C. In untreated tumor bearing controls, the hepatic replacement area was close to 100% in all 3 mice examined (i.e. there was essentially no normal liver tissue detectable in these animals). Animals treated with low dose transposon alone had a mean HRA of 68.3%, and animals that received various doses of SB plasmid had mean HRAs ranging from 12.83 to 35.26%. The group that received 10 μg of SB transposase plasmid was the only group that differed significantly from untreated controls. Despite the striking means, the lack of significant difference observed for the other treated groups was probably due to low sample size and variability among samples.

### Effect of antiangiogenic transposon gene therapy on tumor endothelium and proliferation of colorectal cancer cells

**Figure 6:**
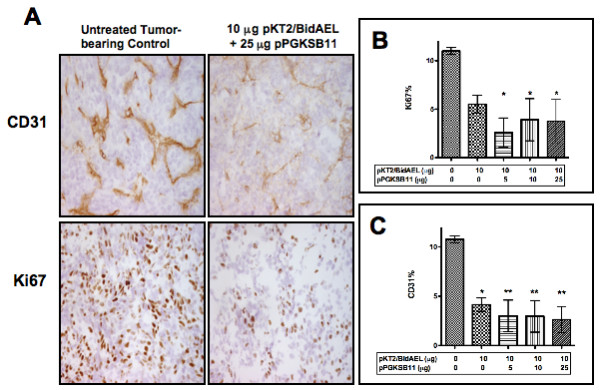
**Immunohistochemical analysis of tumor tissues for cell proliferation and tumor vasculature**. **A**, IHC staining to determine tumor vasculature and proliferation was performed in 5 μm sections on liver. Samples from animals treated with 10 **μ**g transposon plasmid and varying amounts of transposase plasmids were stained using anti-CD31 (tumor vasculature) and anti-Ki67 antibodies (tumor proliferation) and visualized by light microscopy. **B**, Percent positively staining area using Ki67 antibody; **C**, Percent positively staining area using CD31 antibody. Percentage of positively stained areas in respective groups was determined by measuring the area of immunopositivity vs. total tissue area in each of 10 random fields (mean +/- SD). Transposon (pKT2/BidAEL), and transposase (pPGKSB11) plasmid DNA amounts infused into animals are summarized below the graph. *P < 0.05; **P < 0.005

Tumors were evaluated for the effect of low dose Statin AE transposon treatment on proliferation of tumor cells and on tumor vascularization by histomorphometric evaluation of serial immunohistochemistry sections using antibodies directed against Ki67 and CD31, respectively (Figure 6A, B, and 6C). The highest level of tumor cell proliferation (9.6%) was observed in the tumor bearing untreated group, with significantly lower mean proliferation indices (2.6-4.8%) seen in animals that received Statin AE transposon plasmid with or without SB transposase plasmid (Figure 6B). Similarly, tumor tissues from tumor bearing untreated mice had the highest index of CD31 positive blood vessels (9%). In comparison, animals that were treated with Statin AE transposon plasmid with or without SB transposase plasmid demonstrated significantly lower mean (3-3.5%) of vessel density (Figure 6C). There was no significant difference between animals treated with vs. without SB transposase plasmid for either Ki67 or CD31 preparations.

### Extended survival of tumor-bearing mice treated with antiangiogenic transposon

**Figure 7:**
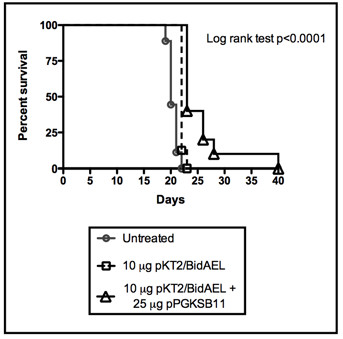
**Survival of mice treated with antiangiogenic transposon**. The Kaplan-Meier plot shows the fraction of animals surviving vs. time after implantation of CT26 cells at day 0 with subsequent hydrodynamic injection of SAE transposon with or without SB transposase-encoding plasmid on day 3 (8 mice per group). Differences in survival between groups was evaluated by log-rank analysis. *P < 0.005

An additional cohort of tumor bearing animals was tested to determine the effect of SB transposase plus statin AE transposon administration on survival. Animals were implanted with 5 × 10^4 ^tumor cells intrasplenically as described in Materials and Methods and then 3 days later, injected hydrodynamically with 10 μg SAE transposon with or without 25 μg SB11 transposase plasmid. The animals were imaged weekly, demonstrating high level luciferase expression and verifying effective hydrodynamic gene delivery (data not shown). All untreated animals, as well as animals treated with transposon alone, succumbed to tumor and were euthanized by day 22 and 23 respectively (Figure 7). In contrast, survival was significantly extended (p < 0.0001), to day 40 in some cases, for animals that were treated with both transposon and transposase encoding plasmids. Tumor burden was evaluated at time of sacrifice, verifying the growth of hepatic metastases (not shown). We conclude that inhibition of CT26 tumor growth brought about by coinfusion of SAE transposon plus SB transposase encoding plasmid confers increased survival in tumor-bearing animals.

## Discussion

StatinAE gene transfer using a *Sleeping Beauty *transposon vector resulted in antitumoral effects in an animal model of colorectal cancer metastatic to the liver. Significant tumor growth inhibition was seen in mice injected with a high (25 μg) dose of antiangiogenic transposon plasmid, in which co-injection of the SB transposase did not achieve any further tumor regression. In contrast, antitumor activity of the Statin AE transposon administered at a lower dose (10 μg) was dependent on co-infusion of SB transposase-encoding plasmid. These results suggest that antiangiogenic gene therapy using the SB non-viral transposon system has the potential to be an effective treatment for colorectal cancer metastatic to the liver, and that this process is dependant on the transposition process under transposon dose-limiting conditions.

Although antiangiogenic proteins show great promise in preclinical cancer models, their effectiveness in the clinic, especially when administered alone, has been limited [34-36]. The effect of VEGF on tumor and vascularization has been studied by many groups, demonstrating it's pivotal role in increasing vascular permeability, tumor growth and metastasis [37,38]. In pre-clinical studies, VEGF targeting strategies have demonstrated significant antivascular effects and tumor growth inhibition. Although several drugs and small molecule inhibitors of VEGF are being clinically tested, their efficacy as monotherapeutic agents in advanced stage disease has been discouraging, due to short half-lives and dose limiting toxicities [39]. However, there is a significant survival benefit when VEGF-targeted therapy is combined with standard chemotherapy for metastatic colorectal cancer [36]. Also, most inhibitors of angiogenesis exert their effect against newly formed blood vessels rather than existing vasculature. The inability of these agents to completely eradicate disease presents a serious challenge for patients with advanced malignant tumors. For most angiogenesis inhibitors, tumor growth resumes after cessation of therapy [39]. This necessitates administration of high doses and prolonged treatment with antiangiogenic proteins to obtain a sustained therapeutic effect.

Delivery of gene sequences encoding recombinant inhibitors of angiogenesis potentially allows for localized and sustained expression, which could reduce the risk of systemic toxicity and perhaps increase efficacy. Other advantages include the possibility of expressing multiple antiangiogenic gene products that act via different mechanisms, thereby increasing the effectiveness of the therapy. The antitumor effects of introducing gene sequences encoding endogenous anti-angiogenic proteins endostatin and angiostatin have been characterized in several studies, and their synergistic effects when used in combination (either in individual or separate vectors) is well documented [29-31,40]. We therefore investigated an angiostatin-endostatin fusion protein previously shown to confer potent antitumor effects in a subcutaneous melanoma model in vivo [32].

The integrating Sleeping Beauty (SB) transposon system circumvents the primary limitation of non-viral plasmid based gene transfer, i.e. the transient duration of gene expression that fails to give way to long-term expression. SB-mediated transposition has been shown to occur in a variety of cultured cell types, in zebrafish [41] and mouse embryos and germ cells [42], in human primary blood lymphocytes [26], and in mouse somatic tissues, including the lung and the liver [25,43-46]. SB-mediated transposition in mouse liver has been verified in several laboratories by recovery and sequencing of transposon-chromosome junction sequences [22,25,44,47-52].

SB has also been used successfully to deliver antitumor genes to neoplastic tissue. Ohlfest et al successfully used the SB transposon system to deliver a cocktail of antiangiogenic genes to human glioblastoma xenografts in mice, subsequently observing increased survival and sustained regression of tumor [27]. Wu et al compared the efficacy of interferon-gamma immunogene therapy using non-integrating plasmid vectors vs SB plasmid vectors in a syngeneic glioma model. Only animals co-injected with SB transposase plasmid exhibited prolonged expression of interferon-gamma and a significant increase in survival (3 weeks), while expression in animals treated with transposon plasmid alone was undetectable after 1 week [53].

While hydrodynamic tail vein injection has been used very effectively for delivering DNA to liver tissue in rodents, extending this technique to humans is still implausible. However, several laboratories have reported the use of balloon occlusion catheters for successful gene delivery to the liver in large animals. Delivery of DNA into occluded rabbit liver under X-ray guidance [54], into the left lateral lobe of pig liver by catheterization and occlusion of the portal vein [55], and to the whole liver of pig and dog via the inferior vena cava (IVC) with double balloon occlusion above and below the IVC-hepatic vein conjunctions [56,57] have been reported. Liu et al have developed a device that uses high pressure from a gas cylinder and a computer-controlled switch to drive and regulate DNA injection in pigs [58]. Use of this device, combined with vessel occlusion and image guided catheterization to achieve site specificity, was found to provide effective gene delivery [59]. Overall, these results from large animal studies demonstrate that modifications of the hydrodynamic technique can potentially be applied to humans.

Our results demonstrate that animals injected with both transposon and transposase-encoding plasmids survive significantly longer than untreated control animals, or animals treated with transposon plasmid alone. However, transgene expression declines over time, leading to eventual emergence of tumor metastases. Transgene-specific immune responses, both humoral and cell-mediated, have been described. Aronovich et al have shown that prolonged expression of ß-glucuronidase after SB transposon-mediated delivery elicited an immune response against transgene-expressing cells, which were subsequently eliminated (22). Lutzko et al have characterized humoral immune responses against transgene products by ELISA, and have demonstrated cellular immune responses using lymphocyte proliferation assays [60]. Relative persistence of transgene expression is also mouse-strain dependant. Injection of recombinant adenovirus expressing human alpha-1-antitrypsin (hAAT), resulted in persistent, circulating levels of hAAT in C57Bl/6 mice, while Balb/c mice rapidly neutralized the transgene product [61]. Hodges et al have shown that hydrodynamic injection of CpG replete, supercoiled human Factor IX encoding plasmid in Balb/c mice resulted in loss of transgene expression after 3-4 weeks, while therapeutic levels of Factor IX expression were maintained in mice that received CpG depleted plasmid [62]. Any or all of these factors could account for the decline in gene expression observed in our study. Modulation or counteraction of the immune response is therefore essential in maintaining sustained transgene expression and continued suppression of tumor metastases.

Here, we have shown that the SB transposon system can be used to successfully deliver antiangiogenic genes and inhibit colorectal tumors metastatic to the liver. We observed that treatment with a high dose of antiangiogenic transposon plasmid appeared to be effective with no requirement for co-delivery of SB transposase. However, antitumor effectiveness of Statin-AE transposon when administered at low dose (10 μg) was dependent on SB transposase. Since transposon delivery in large animals and in humans is likely to be infrequent, the low dose treatment is considered to be more representative of the low level of gene transfer that is to be expected in a clinical setting. In this case, co-delivery of the SB transposase encoding plasmid is required, thereby enabling sustained expression of the therapeutic antiangiogenic transgene through transposition, and thus continued inhibition of tumor growth.

## Conclusions

Patients who would benefit the most from antiangiogenic gene therapy may be those with early metastatic disease or even preoperative or perioperative disease [10,11]. In this study, we have shown significant inhibition of liver metastases using the SB transposon system. However, complete eradication of tumor was not seen in any of the treated groups. Clinically, antiangiogenic gene therapy may be most well suited for use as an adjuvant therapy when administered along with existing treatment options of surgery and/or chemotherapy [8,9]. This combination therapy may facilitate the use of low dose chemotherapy in patients. Future preclinical studies will evaluate transposition and animal survival in combination therapy of SB with other cytoreductive treatments.

## Methods

### Plasmids and cell lines

The murine CT26 colon carcinoma cell line (ATCC, Manassas, VA) was routinely maintained in RPMI 1640 medium supplemented with 10% fetal bovine serum and 1% penicillin/streptomycin/fungizone. SB transposon and transposase containing plasmids were constructed using standard molecular cloning techniques. The transposon plasmid containing the bidirectional promoter (pKT2/LuBiG) is described in Multhaup et al [63]. Statin AE was cloned as an *EcoR*I-*Not*I fragment from pT2/CAE [27] into plasmid pKT2/PGCL to generate plasmid pKT2/BidAEL. The SB transposase plasmid pPGK-SB11 consists of the phosphoglycerate kinase promoter regulating transcription of the SB11 transposase as described previously [49].

### Establishment of hepatic tumors

Female Balb/c mice (6-8 weeks of age) were obtained from NIH (Frederick, MD) and maintained under specific pathogen-free conditions. All animals were treated according to the *NIH Guidelines for Animal Care *with approval of the IACUC of the University of Minnesota. Exponentially growing CT26 colon carcinoma cells were harvested by trypsinization, suspended in Hanks' balanced salt solution, and 1 × 10^5 ^viable cells (as determined by trypan blue exclusion) in a total volume of 200 μl were injected intraspenically as previously described [64]. Briefly, animals were administered 0.2 ml of an anaesthetization cocktail consisting of ketamine HCl (8 mg/ml; Phoenix Scientific, St. Joseph, MO), acepromazine maleate (0.1 mg/ml; Phoenix Scientific), and butorphanol tartarate (0.01 mg/ml; Fort Dodge Animal Health, Overland Park, KS). A peritoneal incision was made to expose the spleen. Cells were delivered directly to the spleens of recipient animals, and a sterile cotton tip applicator was used to apply pressure over the injection site for 3 minutes. A splenectomy was subsequently performed to remove the primary site of tumor inoculation, thus confining tumor formation to the liver. The incision was closed with staples and the animal allowed to recover.

### Hydrodynamic delivery of transposon and transposase encoding plasmids

3 days following tumor cell implantation, 8 animals per experimental group were weighed and administered 0.03 ml of an anaesthetization cocktail as described above. Lactated Ringers solution was added to plasmid DNA to bring the total volume (in mL) equivalent to 10% of the total body weight (in grams) for each mouse. The plasmid DNA solution was then injected rapidly through the tail vein of the animal in a period of 4-8 seconds. Animals that did not receive the injection in less than 8 seconds were omitted from the study. Injected animals were placed on a heating pad and monitored until recovery from anesthesia [65].

### In vivo luciferase imaging

Animals were anaesthetized using a sedative dose (400 μl i.p. for a 25 gram mouse) of the anaesthetization cocktail described above, followed by intraperitoneal injection of 100 μl of luciferin substrate (28.5 mg/ml; Caliper Life Sciences, Hopkington, MA). Five minutes following luciferin injection, mice were imaged for 1 second, using an intensified CCD camera (Xenogen Corporation, Alameda, CA). Raw values were recorded as photons of light emitted per second [66].

### Animal necropsy and immunohistochemistry

Animals were sacrificed 21 days post-tumor seeding, or at the time of declining health in the survival study. Animals were euthanized according to the University of Minnesota IACUC-approved protocols. The date of euthanasia was recorded as the end day of survival and autopsy was subsequently performed. Liver weights and tumor nodule counts were recorded. Intact livers from all groups except the nontumor bearing control group were snap frozen in 2-methylbutane and stored in liquid nitrogen. Frozen tissue blocks were sectioned at 5 microns, mounted on slides and stored at -80°C until use, at which time they were allowed to come to room temperature, fixed in acetone for 10 min, and either stained routinely with hematoxylin and eosin or immunostained using antibodies directed against CD-31 or Ki67. For this procedure, endogenous peroxidase activity was blocked with 0.3% H_2_O_2 _for 15 minutes. Undiluted avidin and biotin were applied for 15 minutes each to block non-specific biotin (DAKO X0590). In addition a protein serum block was applied for 15 minutes. The appropriate primary antibody (rat anti-mouse CD 31; BD Pharmingen Cat #550274 or rat anti-mouse Ki-67; Dako Cat # M7249) was applied at a dilution of 1:100 (CD31) or 1:25 (Ki-67) for 60 minutes. The secondary antibody (biotinylated anti-rat 1:300; Vector Laboratories Cat # BA-4001) was applied for 30 minutes followed by undiluted streptavidin-HRP (Dako Cat # K1016). The brown reaction product was detected using 3,3'diamino-benzidine (Dako Cat # K3466). Sections were counterstained with Mayer's hematoxylin. For negative control sections, normal rat serum was substituted for the primary antibody.

### Histomorphometry

The H&E stained tissue sections were imaged using a 1× objective and the immunohistochemical sections were imaged using a 20× objective. The 1× images were captured with a Nikon E800 microscope and a Nikon DXM 1200 digital camera (Nikon Instruments Inc., Tokyo, Japan) and included the entire area of tissue. The 20× images were captured with an Olympus BX40 (Olympus, USA) and a SPOT Insight digital camera (Diagnostic Instruments Inc., Sterling Heights, IL) and were taken only within tumor tissue. Measurement of images (1X) of the H&E stained sections were used for morphometric evaluation of % tumor area using Image-Pro Plus 6.2 (Media Cybernetics, Silver Springs, Maryland). Tumor area (%) was determined by tracing the outer margin of each metastatic lesion and determining its area, summing the areas of all metastatic lesions in the tissue section, and dividing this sum by the total area of the tissue section.

Images of the immunohistochemistry sections were analyzed using Image-Pro Plus 6.2 (Media Cybernetics, Silver Springs, Maryland). Areas within the tumors that stained positively for Ki-67 or CD31 by IHC were differentiated from negative areas by using the threshold command. 5 randomly selected fields (field size approximately 265,350 square microns) from tumor tissue in each section were evaluated. The total area of tissue as well as the area of immunopositivity for CD-31 or Ki-67, respectively, were measured (μm^2^). The area of immunopositivity was then recorded as a percentage of the total area. Results from the 5 fields were then averaged for each section.

### Statistical analysis

Data were analyzed using GraphPad Prizm 5.0 software (GraphPad Software Inc., San Diego, CA). Statistical analysis of differences between groups was determined using one way analysis of variance (ANOVA), with Tukey's post test analysis. Animal survival was evaluated by the Kaplan-Meier product limit method, comparing differences among animal groups by the log rank test. Differences were considered significant when P < 0.05 for ANOVA.

## Competing interests

R. Scott McIvor is a founder and manager of Discovery Genomics, Inc.

## Authors' contributions

LRB carried out the molecular cloning, experimental design, data acquisition/analysis, and drafted the manuscript. KPP carried out the plasmid hydrodynamic injections, and for animal care. BS performed surgical implantation of tumor cells. AH participated in animal experiments and data collection. JP and CSS carried out immunohistochemical assays and quantitation. DS and SR provided key experimental resources and critically reviewed the manuscript. RSM conceived the study, participated in its experimental design and co-wrote the manuscript. All authors read and approved the final manuscript.
